# An eco-friendly liquid chromatographic analysis of the triple therapy protocol of amoxicillin, metronidazole and vonoprazan for *H. Pylori* eradication: application to combined dosage forms and simulated gastric fluid

**DOI:** 10.1186/s13065-024-01210-6

**Published:** 2024-05-30

**Authors:** Hoda Mahgoub, Marwa A. A. Ragab, Salma Tarek, Hadir M. Maher

**Affiliations:** Department of Pharmaceutical Analytical Chemistry, Faculty of Pharmacy, Alexandria University, El-Messalah, Alexandria, 21521 Egypt

**Keywords:** Amoxicillin, Metronidazole, Vonoprazan, HPLC- DAD, Simulated gastric fluid

## Abstract

*Helicobacter pylori* has a big sway when peptic ulcers are concerned. For its eradication, different protocols have been approved. Among which, the tripartite therapy protocol which embraces vonoprazan as potassium competitive acid blocker in combination with amoxicillin and metronidazole as antibiotics. An environmentally benign HPLC method is addressed in order to simultaneously determine amoxicillin (AMX), metronidazole (MET) and vonoprazan (VPZ) in bulk powder and combined tablet mixture. Full separation of AMX, MET and VPZ is accomplished using C8 column, and a gradient mobile phase system, composed of methanol and phosphate buffer of a pH value of 5. Fine linearity in the concentration ranges 50–600 µg mL^−1^ amoxicillin, 50–400 µg mL^−1^ metronidazole and 10–100 µg mL^−1^ vonoprazan was denoted by the high correlation coefficient (0.9999). The method accuracy and precision are confirmed upon analyzing AMX, MET and VPZ triple therapy not only in their synthetic mixtures and combined tablet mixtures but also in their combined tablet mixtures in simulated gastric fluid. AMX, MET and VPZ triple therapy could be routinely analyzed in QC labs, in case of being co-formulated, using the presented method. Three different assessment tools were adopted revealing the benign environmental impact of presented method.

**Supplementary Information:**

The online version contains supplementary material available at 10.1186/s13065-024-01210-6.

## Introduction

*Helicobacter pylori* (*H. pylori*) bacteria are guilty of causing a wide range of gastrointestinal illnesses, to be specific, chronic gastritis, peptic ulcers, gastric adenocarcinoma, and mucosa-associated lymphoma [1]. Eradication of *H. pylori* infection has been the major concern of health care providers and many treatment regimens have been developed in an attempt to prevent gastric cancer. Despite the resolute endeavors to manage *H. pylori* infection, it is still immensely prevalent worldwide, particularly in developing countries [1].

When it comes to controlling *H. pylori* infection, variant combination therapies have been sought. A proton pump inhibitor (PPI) with the antibiotics clarithromycin and amoxicillin (AMX)/metronidazole (MET) comprises the classic tripartite eradication therapy. This combination represents the first-choice for management of *H. pylori* infection [2, 3] till the past decades where the problem of antibiotic resistance has been explored. The efficacy of using antibiotics in treatment protocols is unfortunately hindered by the global crisis of antibiotic resistance. This problem is highly encountered with clarithromycin, MET, and levofloxacin which is increasing by time [4–6]. As a result, failure to meet the clinical efficacy may direct the physicians towards the increase in the dosage of antibiotics to get the required therapeutic effect. Accordingly, the prevalence of adverse events with development of higher level of tolerance may further complicate the situation. Meanwhile, dual therapy containing PPI and AMX has gained a wide popularity with required treatment outcomes. AMX is a broad-spectrum penicillin antibiotic [7]. The time-dependent pattern of AMX action lead to the necessity of its daily frequent administration up to thrice or four times in order to get high plasma concentration levels above the minimum repressing concentration. Acid suppression is a fundamental pillar in treatment protocols of *H. pylori* treatment regimens by increasing the susceptibility of the bacteria to antibiotics and increasing the chemical stability of some antibiotics, including AMX [8]. Optimum pH for AMX action (above 6) was achieved using high doses of the PPI. In addition, low incidence of developing resistance to AMX, unlike clarithromycin, MET, and levofloxacin, contributes to the efficacy of this antibiotic in eradication protocols with hefty eradication rates of more than 90% [9]. Despite the merits of this binary therapy, eradication rates fluctuates among different studies with reported dependance on the CYP2C19 genotype [10, 11]. However, the use of AMX-containing combinations could be limited by penicillin allergies [12].

Further trials have divulged that potassium-competitive acid blockage can come up with a superior acid suppression compared with PPIs with a rapid onset of action [13, 14]. Moreover, its acid suppression is less affected by the CYP2C19 enzyme genotype compared to PPIs [15]. As a result, this was found advantageous when used with AMX-based combinations. Vonoprazan (VPZ) is the most acclaimed contender belonging to potassium-competitive acid blockers and VPZ -based dual and triple therapies have been approved for *H. pylori* eradication [16, 17].

**Fig. 1:**
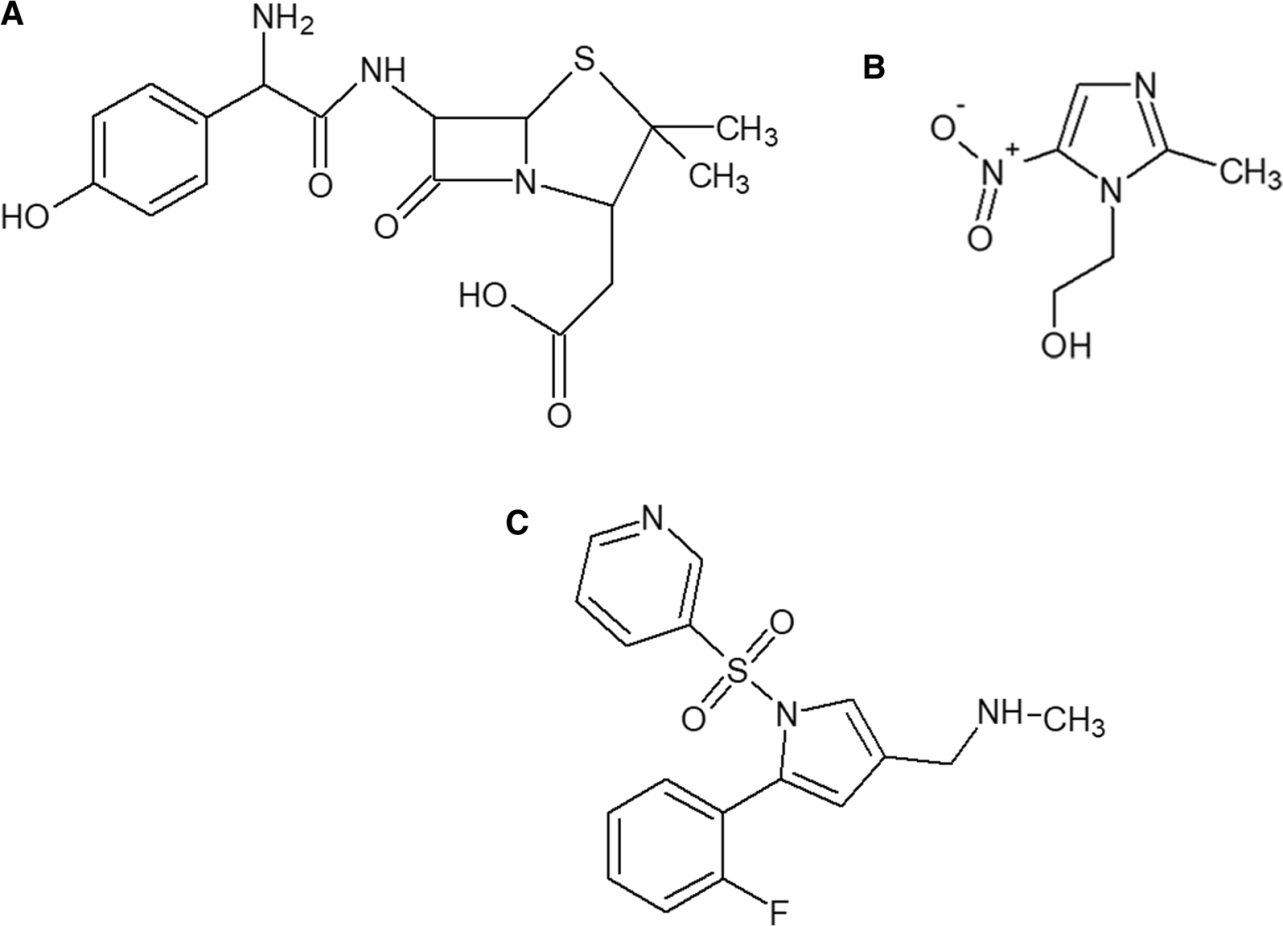
Chemical structures of (**a**) amoxicillin, (**b**) metronidazole and (**c**) vonoprazan fumarate

It has been lately discovered that a tripartite treatment protocol embracing VPZ as a potassium-competitive acid blocker with AMX and MET is efficient in the treatment of *H. pylori* infections as stated in the following protocol: 20 mg VPZ with 750 mg AMX and 250 mg MET twice a day (b.i.d) for 7 days, particularly in cases of clarithromycin resistance [18]. The additive antimicrobial effect of AMX and MET was previously reported. This triple combination of AMX, MET, and VPZ was the concern of our study. Chemical structures of these drugs were provided in Fig. 1. The chemical name of AMX is (2S, 5R, 6R) -6- [[(2R)-2 -amino-2-(4hydroxyphenyl)acetyl] amino]-3,3-dimethyl7-oxo-4-thia-1-azabicyclo [3.2.0] heptane-2-carboxylic acid. MET is (2-Methyl-5-nitro-1H-imidazol-1-yl) ethanol. While VPZ is 1-(5-(2-fluorophenyl)-1-(pyridin-3- ylsulfonyl)-1H–pyrrol − 3-yl)-N-methylmethanamine [19, 20].

Upon reviewing the literature, it has been divulged that binary mixtures of AMX and MET were previously determined using hydrophilic interaction chromatography (HILIC) [21], UV spectroscopy [22, 23] and HPLC [24]. Also, AMX and MET but this time with famotidine (FAM), an HPTLC report for their analysis in simulated gastric juice was found [25]. In addition, VPZ with its related substances analysis was adopted via a HPLC method [20]. UV- spectrophotometry was used for VPZ analysis with aspirin [26]. Moreover, VPZ was analyzed in human plasma by fluorimetry [27] and LC–MS [28].

As far as we know, for AMX, MET, and VPZ ternary mixture, no method has been reported in the literature so far dealing with their determination. Environmentally- benign HPLC method development was the target of the presented study, which is able to determine AMX, MET, and VPZ in bulk powder and in combined-tablet mixtures besides in simulated gastric fluid. The adopted HPLC-diode array detector (DAD) method could be utilized for the regular analysis of AMX, MET, and VPZ in quality control labs augmenting the merits of being simple, accurate, economic, and environmentally friendly. For method greenness appraisal, four variant tools were utilized: the analytical eco-scale (ESA) [29], analytical greenness metric approach and software (AGREE) [30], the green analytical procedure index (GAPI) [31] and the national environmental method index (NEMI) methods [32].

## Experimental

### Instrumentation

The HPLC–DAD system (Agilent Technologies, Santa Clara, CA, USA) consisted of Agilent 1200 Series Quaternary pump G1311A which comprises a solvent cabinet and an Agilent 1200 Series Vacuum Degasser G1322A. Diode Array and Multiple Wavelength detector were used. The LC system is equipped with Agilent 1200 Series Thermostated Column Compartment and autosampler. This system is connected to a computer loaded with Agilent ChemStation Software. LC separation was performed on an Agilent Zorbax Eclipse SB-C8 analytical column (250 × 4.6 mm, 5 µm). For weighing, a Scal-TEC balance model SPB 31, Germany was used. A Julabo sonicator model USR 3, Julabo Labortechnik, GMBH, Germany was used for sonication. A Cyberscan pH meter model 510 was used to adjust the pH of the mobile phase and simulated gastric fluid. Agilent solvent filtration assembly was used for mobile phase filtration, however, samples were filtered using 0.45 μm nylon syringe filters.

### Materials and reagents

Working standard AMX was supplied from Pharco Pharmaceuticals Company, Alexandria, Egypt. Its purity was 99%. However, working standard substance of MET was supplied from Medical Union Pharmaceuticals Company, Ismailia, Egypt with purity 99.6%. Working standard substance of VPZ (as VPZ fumarate) was supplied from Inspire Pharmaceuticals Company, Egypt with purity 99%. Biomox^®^ tablets which are labeled to contain 500 mg AMX per tablet and manufactured by SEDICO, Egypt and Flagyl^®^ tablets which are labeled to contain 250 mg MET per tablet and manufactured by SANOFI, Egypt, both were purchased from local market. Vonaspire^®^ tablets which contain VPZ fumarate and labeled to contain 20 mg VPZ per tablet were manufactured by Inspire, Egypt and also was purchased from the local market. Sigma-Aldrich Chemie GmbH, Swizterland Germany provided HPLC- grade methanol. Sodium hydroxide pellets, sodium dihydrogen orthophosphate, and hydrochloric acid 37% were purchased from El-Nasr Chemical Industry Company, Egypt and all were of analytical grade. Extra pure sodium chloride was supplied from Oxford Lab Chem, Navghar, Vasai, Maharashtra, India. High purity distilled water was used.

### General procedure and construction of calibration graphs

#### Preparation of stock, diluted stock, and working solutions

Separate stock solutions 2000 µg mL^−1^ of each of AMX and MET and 1000 µg mL^−1^ of VPZ were prepared in HPLC-grade methanol. Then diluted stock solutions 1000 µg mL^−1^ of each of AMX and MET and 400 µg mL^−1^ VPZ were prepared by diluting appropriate aliquots of the stock solutions with HPLC-grade methanol into volumetric flasks of 50 mL capacity. The diluting solvent (methanol: 30 mM phosphate buffer pH 5) (5:95 v/v) was used for diluting proper aliquots of the diluted stock solutions in order to obtaining final working solutions into a set of 10-mL volumetric flasks to reach the concentration ranges of 50- 600, 50–400, and 10- 100 µg mL^−1^ for AMX, MET, and VPZ, respectively.

#### Chromatographic conditions and construction of calibration graphs

HPLC separation was achieved using an Agilent Zorbax Eclipse SB-C8 analytical column (250 × 4.6 mm, 5 µm) with a mobile phase composed of methanol (solvent A) and phosphate buffer of 30 mM concentration and pH of 5 (solvent B), established in a gradient elution mode,. The gradient elution program used was outlined in supplementary table S1. A membrane filter of pore size 0.45 µm (Millipore, Milford, MA, USA) was used for mobile phase filtration. The flow rate was 1.0 mL min^−1^ and 20-µL injection volume was applied. Setting the photodiode array detector at 230 nm for AMX and VPZ quantitation and 320 nm for MET quantitation was an important step to perform the analysis. A mixture of solvent A: solvent B (5: 95 v/v) was used as a diluting solvent for samples.

Each working solution was filtered using 0.45 µm disposable filters. Then three injections of 20-µL volume of each AMX, MET and VPZ concentrations were chromatographed. Plotting the AMX, MET and VPZ peak areas versus the correlative concentrations was done in order to obtain their calibration graphs and compute their regression equations.

### Applications of the proposed method

#### Assay of the individual tablets

A total of 20 tablets of each of Biomox^®^ 500 mg, Flagyl^®^ 250 mg, and Vonaspire^®^ 20 mg were weighed and finely powdered. Powders were weighed exactly to obtain amounts equivalent to 500 mg AMX, 500 mg MET, and 250 mg VPZ, then the weighed amounts were transferred into separate 250-mL volumetric flasks with the aid of 100 mL HPLC- grade methanol. Ultrasonic bath was used for sonication of the flasks contents for 30 min then the volumes were made up using the same solvent to obtain 2000 µg mL^−1^ stock solutions of each of AMX and MET, and 1000 µg mL^−1^ of VPZ.

After filtration, diluted stock solutions were prepared as described in Sect. "Preparation of stock, diluted stock, and working solutions" to obtain 1000 µg mL^−1^ of each of AMX and MET and 400 µg mL^−1^ of VPZ. Each of AMX, MET and VPZ was appropriately diluted into a set of 10-mL volumetric flasks to reach 300 µg mL^−1^ AMX, 300 µg mL^−1^ MET and 100 µg mL^−1^ VPZ. Then the procedure was adopted as expounded under the construction of the calibration graphs in Sect. "General procedure and construction of calibration graphs".

#### Assay of laboratory-prepared combined tablet mixtures

Two tablet- mixture solutions of the three drugs were obtained upon diluting suitable aliquots of the above mentioned diluted stock solutions in Sect. "Assay of the individual tablets" with the diluting solvent to reach 600 µg mL^−1^ AMX, 200 µg mL^−1^ MET and 16 µg mL^−1^ VPZ for the first tablet- mixture solution which represents a recommended dosage ratio for *H. pylori* treatment. However, to extend the method applicability, a second tablet- mixture solution with different drugs ratios was prepared to contain 100 µg mL^−1^ AMX, 200 µg mL^−1^ MET, and 100 µg mL^−1^ VPZ. Then the procedure was adopted as shown under the construction of the calibration graphs in Sect. "General procedure and construction of calibration graphs".

#### Assay of laboratory-prepared combined tablet mixtures in simulated gastric juice

In order to prepare the simulated gastric fluid which will be used throughout the study, firstly, 2 g NaCl was dissolved in about 900 mL distilled water, followed by tuning the pH value to reach 1.2 ± 0.1 with the aid of 0.1N HCl. At last, distilled water was used to made up the volume to 1000 mL [33, 34]. The same amounts of the dosage forms stated in Sect. "Assay of the individual tablets" were weighed and brought into separate 250-mL volumetric flasks with the aid of 100 mL of the prepared simulated gastric fluid. Ultrasonic bath was used for the sonication of the flasks content for 30 min then the volumes were made up using the mentioned simulated gastric fluid.

After filtration, HPLC- grade methanol was utilized to prepare diluted stock solutions as described in Sect. "Assay of the individual tablets" in order to obtaining 1000 µg mL^−1^ of each of AMX and MET, and 400 µg mL^−1^ of VPZ. Then dilutions were made using the diluting solvent in order to prepare the same two tablet- mixture solutions of the three drugs mentioned in Sect. "Assay of laboratory-prepared combined tablet mixtures". The same procedure was followed as described under the construction of calibration graphs in Sect. "General procedure and construction of calibration graphs".

## Results and discussion

### Optimization of the chromatographic conditions

Different experimental parameters were carefully studied and optimized so as to attain good separation of AMX, MET, and VPZ with symmetric peak shape, fine resolution, and reproducible retention time. In this respect, it is important to mention that VPZ was formulated as fumarate salt. Thus, it was eluted as two peaks, the unretained fumarate peak being co-eluted with the solvent front and another peak corresponding to VPZ base which was used for the assay of the parent drug. Since the hydrophilic nature of AMX contributes to its rapid elution, being the least retained compound among the three analytes, the coelution of AMX with the fumarate salt constitutes an additional challenge in the determination of such mixture.

#### Stationary phase

**Fig. 2:**
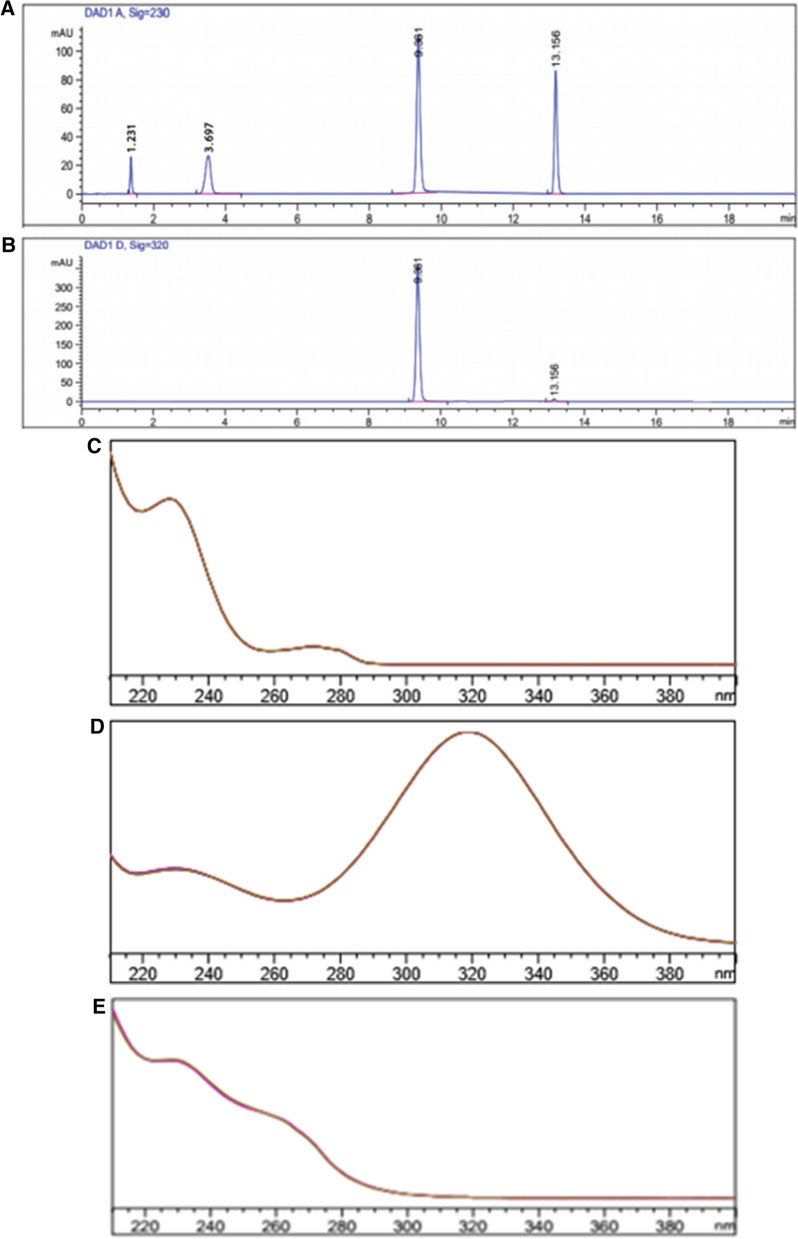
Typical HPLC chromatograms of a solution containing 100 µ  mL^−1^ AMX, 200 µg mL^−1^ MET and 100 µg mL^−1^ VPZ (t_R_ = 3.70, 9.35 and 13.16 min, respectively) scanned at 230 nm for AMX and VPZ (**a**) and 320 nm for MET (**b**) and with their purity profiles: AMX (**c**), MET (**d**) and VPZ (**e**) obtained from DAD connected to HPLC

The effect of different column types (packing material) on the separation of AMX, MET, and VPZ from each other was studied. The investigated separating columns were Agilent Zorbax Eclipse SB-C18 analytical column (250 × 4.6 mm, 5 µm) and Agilent Zorbax Eclipse SB-C8 analytical column (250 × 4.6 mm, 5 µm). C8 column was adopted for this study as a conducted comparison between: C18 column –obtained chromatograms (supplementary figures S1a and b), and those of C8 column- obtained chromatograms (Fig. 2a and b). The comparison revealed the superiority of the C8 over the C18 as the first gave better peak sharpness and symmetry for AMX and VPZ peaks.

#### Type and ratio of organic modifier

Each of acetonitrile and methanol have been investigated as organic modifiers through the optimization of the HPLC method. Unlike methanol, using acetonitrile in different ratios, no separation was achieved since the three drugs were overlapped so methanol was considered optimum and was picked out so as to chromatographically separate AMX, MET and VPZ with reasonable retention times. A mixture of methanol (solvent A) and sodium dihydrogen phosphate of 30 mM concentration and pH value of 5 (solvent B) was adopted as the mobile phase for this study. In the early trials, different ratios of the organic modifier and the aqueous phase were tried in the isocratic mode. Unfortunately, all the tried isocratic systems were unable to resolve AMX, MET, and VPZ from each other (supplementary Table S2). Therefore, switching to the gradient mode was mandatory in order to separate AMX, MET and VPZ. Two gradient programs were tried. The first one started with 10% methanol (by volume) from 0 to 3.2 min then it was ramped up to reach 50% at 8 min and remained steady till 13 min then it was returned to 10% at 18 min. The second began with 5%, methanol (by volume) from 0 to 6 min then it ramped up to get as far as 50% at 10 min and remained steady till 15 min then it was returned to 5% at 20 min. The second gradient program with lower methanol content as starting ratio prevented the overlapping between AMX and fumarate peaks. Besides, the k' value of AMX peak was in the accepted range with better separation between MET and VPZ. The second gradient program resulted in the finest balance between adequate resolution, rational retention times, and tolerable peak asymmetry. AMX, MET, and VPZ were separated at 3.70, 9.35 and 13.16 min, respectively which are reasonable retention times (Figs. 2a, b).

#### Selection of phosphate buffer pH

At least 2 pH units difference, between the value of the compounds of concern and the chosen pH, is mandatory (pKa of AMX = 2.4, 7.4 and 9.6, pKa of MET = 2.5 and pKa of VPZ = 9.6) [35, 36]. Therefore, pH 5 was chosen as intermediate pH providing peak sharpness and symmetry.

#### The ionic strength of the selected buffer

A study was conducted regarding the influence of the ionic strength of the buffer of choice using 10, 30 and 50 mM phosphate buffer. It was discovered that the best k' of AMX and the best sharpness of MET peak were brought by using 30 mM phosphate buffer so this buffer concentration was selected.

#### Detection wavelength

The photo diode array detector was adjusted at 230 nm for quantitation of AMX and VPZ, and 320 nm for quantification of MET, in order to maximize the assay sensitivity. Figure 2c, d and e show the UV-absorbance spectra over the range 200–400 nm of AMX, MET, and VPZ, respectively.

### System suitability

The numerical values of the system suitability parameters embracing capacity factor (k' > 2), number of theoretical plates (N > 2000), selectivity (α), resolution (Rs > 2) and asymmetry factor (As ≤ 2) are outlined in supplementary table S3. These values indicated that the separation of AMX, MET and VPZ ternary mixture was efficient and selective [37].

### Validation of the proposed method

The ICH guidelines for method validation were adopted for validation of the developed HPLC method [38].

#### Linearity

A series of different concentrations of AMX, MET and VPZ were prepared in order to assess the linearity. Calibration graphs were created by plotting peak area values versus concentrations and they showed linear relationships for AMX, MET and VPZ (supplementary Figures S2a, b and c). The adopted concentration ranges were 50–600 (AMX), 50–400 (MET), and 10–100 (VPZ) μg mL^−1^ as shown in Table 1. The linear least squares regression was used in order to appraise the different statistical parameters: slopes, intercepts and correlation coefficient, Table 1. The high values of the correlation coefficients (r ≥ 0.9999) with high F values (≥ 2624.36) and the small significance F values (≤ 3.81 × 10^–4^) indicated the good linearity of the calibration graphs. Low RSD% of the slope (S_b_%) was obtained (≤ 2%) as it was 0.53, 1.65, and 1.96 for AMX, MET, and VPZ, respectively [39].

**Table 1:** Analytical parameters for determination of AMX, MET and VPZ ternary mixture using the proposed HPLC–DAD method

Parameters	Drugs		
	AMX	MET	VPZ
Wavelength (nm)	230	320	230
Concentration range (µg mL^−1^)	50–600	50–400	10–100
Regression equation	Y = 51.47 + 12.57x	Y = 241.11 + 22.36x	Y = 88.88 + 18.89x
Intercept (a)	51.47	241.11	88.88
Sa^a^	25.49	94.89	40.39
Slope (b)	12.57	22.36	18.89
Sb^b^	6.61 × 10^–2^	0.37	0.37
RSD% of slope	0.53	1.65	1.96
Correlation coefficient (r^2^)	0.99998	0.9999	0.9999
Sy/x^c^	29.43	107.23	51.03
F^d^	137,431.13	15,016.82	2624.36
Significance F	7.28 × 10^–6^	6.66 × 10^–5^	3.81 × 10^–4^
LOD^e^ (µg mL^−1^)	3.7	5.5	2.6
LOQ^f^ (µg mL^−1^)	12.4	18.2	8.5

^a^Standard deviation of the intercept

^b^Standard deviation of the slope

^c^Standard deviation of the residuals

^d^Variance ratio, equals the mean of squares due to regression divided by the mean of squares about regression (due to residuals)

^e^*LOD*  Limit of detection

^f^*LOQ*  Limit of quantitation

#### Detection and quantitation limits

Signal-to-noise ratio of 3:1 and 10:1were used to estimate the limits of detection (LOD) and quantitation (LOQ), respectively. The small values of LOD (3.7, 5.5 and 2.6) μg mL^−1^ and LOQ (12.4, 18.2 and 8.5) μg mL^−1^ for AMX, MET and VPZ, respectively as presented in Table 1.

#### Accuracy

It was evaluated by analyzing AMX, MET, and VPZ in three different laboratory-prepared mixtures (supplementary Table S4). Satisfactory recoveries (98–102%) and small values of relative errors (E_r_%) of − 0.84, − 1.16, and 0.73 for AMX, − 1.94, − 0.23, and − 1.65 for MET and 1.50, − 0.3 and − 0.3 for VPZ in mixtures 1, 2, and 3, respectively were obtained which indicated the high accuracy of the proposed methods.

#### Precision

AMX, MET and VPZ laboratory-prepared mixtures were analyzed in three replicates either on the same day for intra-day precision (repeatability) or on three consecutive days for intermediate precision (inter-day precision). The RSD% values did not exceed 2% as they were 0.50, 1.14, and 0.92 for AMX, 1.53, 1.47, and 1.34 for MET and 0.22, 0.76, and 1.74 for VPZ in mixtures 1, 2 and 3, respectively for intra-day measurements. As for the inter-day measurements, the RSD% values also did not exceed 2% as they were 0.34, 1.43, and 0.25 for AMX, 1.61, 0.95, and 1.72 for MET and 0.60, 1.16, and 0.77 for VPZ in mixtures 1, 2 and 3, respectively. These results appear in supplementary table S4 denoting the acceptable precision provided by the proposed HPLC- DAD.

#### Robustness

Method parameters were subjected to slight changes in order to verify the method robustness such as: mobile phase ratio (± 2%), detection wavelength (± 2 nm) and pH of buffer (± 0.2 pH units). These variations showed significant influence neither on the peak area nor the retention time of the analytes. RSD% did not outstrip 2% as it was 1.23, 0.30 and 0.55 for AMX, 0.98, 1.05 and 1.37 for MET and 1.17, 0.63 and 0.30 for VPZ, respectively (supplementary Table S5).

#### Specificity

**Fig. 3:**
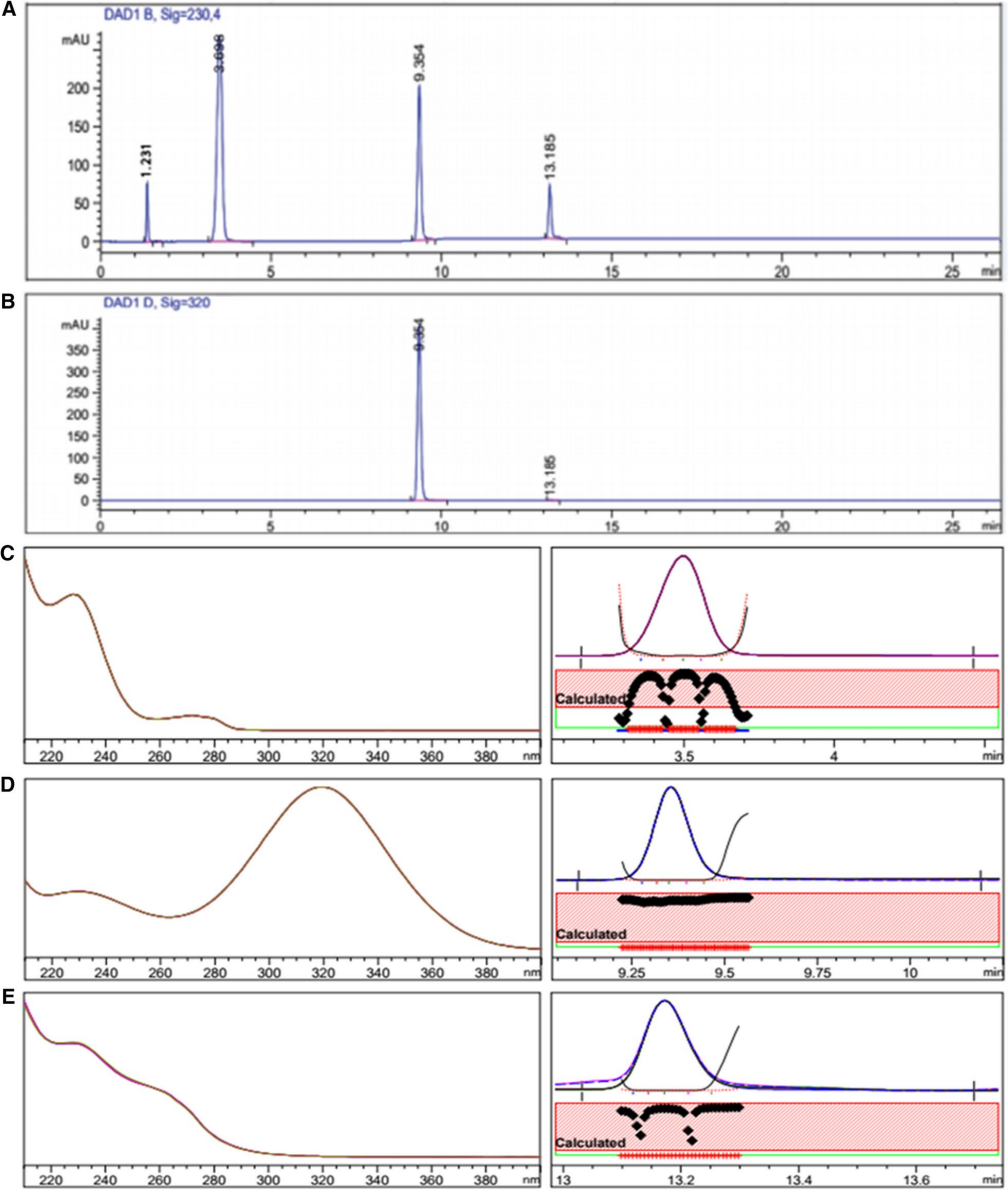
Typical HPLC chromatograms of a laboratory prepared mixture solution of the three dosage forms containing 600 µg mL^−1^ AMX, 200 µg mL^−1^ MET and 16 µg mL^−1^ VPZ in methanol, representing the recommended doses for *H.pylori* treatment (t_R_ = 3.57, 9.35 and 13.16 min, respectively) scanned at 230 nm for AMX and VPZ (**a**) and 320 nm for MET (**b**) with their purity plots and profiles AMX (**c**), MET (**d**) and VPZ (**e**)

The method specificity was verified by analyzing AMX, MET and VPZ laboratory-prepared combined tablet mixtures. No peaks of the inactive ingredients of the preparations were observed (Figs. 3a–e). The peak purity was verified by the aid of DAD.

#### Stability of solutions

Stock and diluted stock solutions of MET and VPZ, prepared in methanol were found to be stable and could be kept in refrigerator for at least one weak but those of AMX were unstable so they were freshly prepared. The working standard solutions besides sample solutions were stable for 5 h at room temperature. The stability was authenticated by chromatographic analysis and monitoring the respective retention time and peak area.

### Applications of the proposed method

#### Assay of the individual drugs in their commercial tablets

AMX, MET and VPZ commercial tablet dosage forms were analyzed by the proposed HPLC–DAD method; each for its corresponding drug; AMX in Biomox^®^ 500 mg, MET in Flagyl^®^ 250 mg, and VPZ in Vonaspire^®^ 20 mg. The prepared final dilution of each drug stated in Sect. "Assay of the individual tablets" was analyzed and the mean % recovery ± SD (n = 3) was calculated from the correlated calibration graph of each drug. The values were satisfactory as they were 99.65 ± 0.27, 98.36 ± 1.33, and 101.00 ± 0.39 for AMX, MET and VPZ, respectively.

#### Assay of laboratory-prepared combined tablet mixtures in methanol

Tablets of the AMX, MET and VPZ are prescribed to be co-administered in the treatment regimen of *H. pylori*. Their combined- tablet mixture solutions were laboratory prepared in HPLC-grade methanol in the same ratio prescribed in therapeutic regimens and analyzed by the proposed HPLC–DAD method. Successful results were obtained and outlined in Table 2. Figures 3a, b show the separation of the combined -tablet mixture solution.

**Table 2:** Assay results for the determination of AMX, MET and VPZ in their laboratory-prepared combined dosage form in methanol and in simulated gastric fluid using the proposed HPLC method (n = 3)

Medium		Nominal value (μg mL^−1^) in prepared Combined dosage form			Mean %recovery ± SD^a^			RSD (%)^b^		
		AMX	MET	VPZ	AMX	MET	VPZ	AMX	MET	VPZ
Methanol	MIX 1	600	200	16	99.42 ± 0.92	100.39 ± 0.89	101.15 ± 0.42	0.93	0.89	0.42
	MIX 2	100	200	100	99.92 ± 0.43	99.62 ± 0.47	100.09 ± 0.84	0.43	0.47	0.84
Simulated gastric fluid	MIX 1	600	200	16	99.63 ± 0.53	98.64 ± 0.47	101.32 ± 0.93	0.53	0.48	0.92
	MIX 2	100	200	100	100.52 ± 1.08	100.25 ± 0.89	101.02 ± 0.27	1.07	0.89	0.27

^a^ mean % recovery for three determinations ± standard deviation (n = 3)

^b^ relative standard deviation

#### Assay of laboratory-prepared combined tablet mixtures in simulated gastric juice

**Fig. 4:**
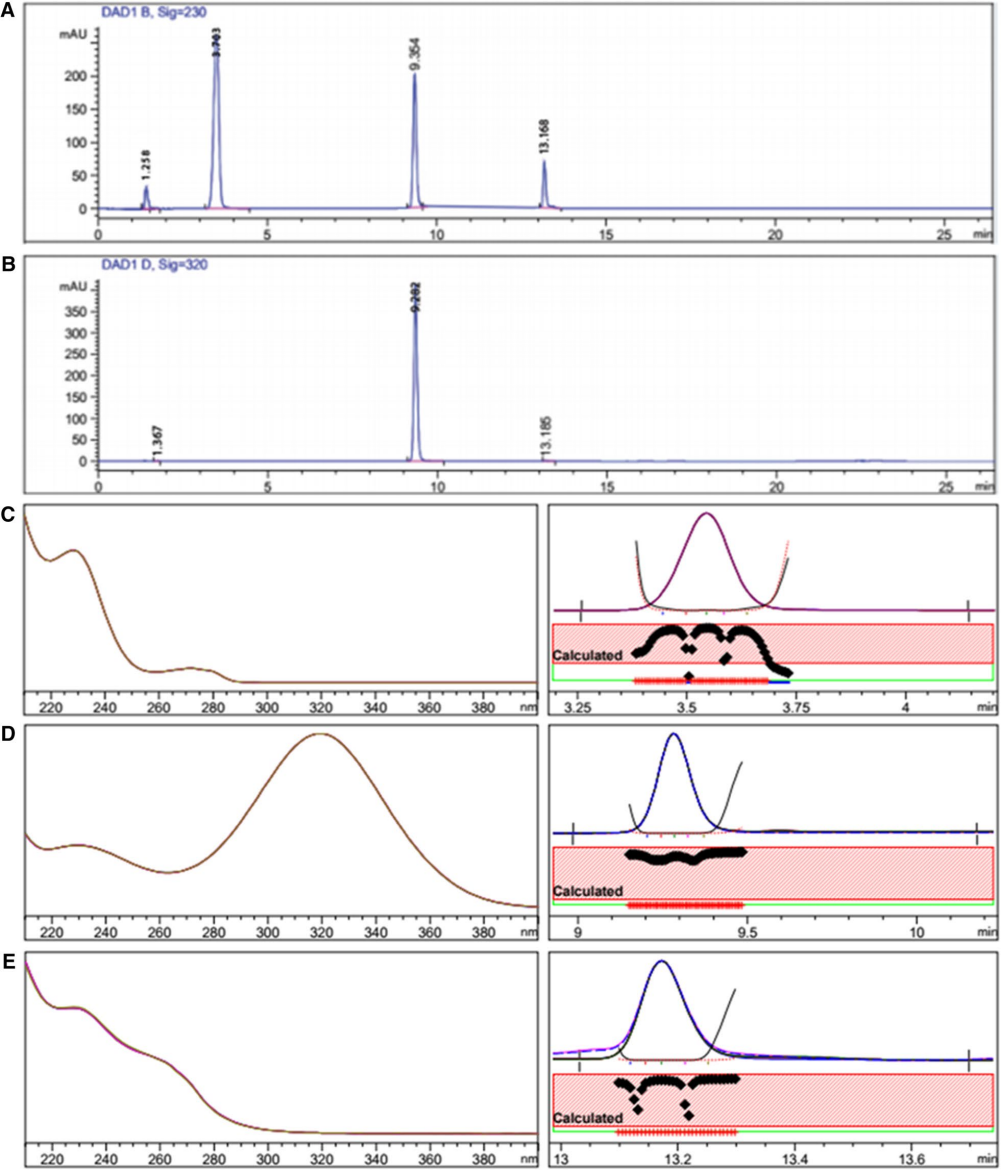
Typical HPLC chromatograms of a laboratory prepared mixture solution of the three dosage forms containing 600 µg mL^−1^ AMX, 200 µg mL^−1^ MET and 16 µg mL^−1^ VPZ in simulated gastric fluid, representing the recommended doses for *H. pylori* treatment (t_R_ = 3.65, 9.34 and 13.17 min, respectively) scanned at 230 nm for AMX and VPZ (**a**) and 320 nm for MET (**b**) with their purity plots and profiles AMX (**c**), MET (**d**) and VPZ (**e**)

It is critical to highlight that the therapeutic agents prescribed in the treatment of gastrointestinal disorders are usually determined in simulated gastric conditions. A previous research work conducted by our research team [25] reported the significance of AMX determination in simulated gastric juice. The same work also described the increase in MET transfer as a result of increase in acid secretion. In this study, combined- tablet mixture solutions were prepared in simulated gastric fluid to investigate the applicability of the proposed HPLC–DAD method for the simultaneous analysis of the three drugs upon the existence of gastric fluid. No interference from the simulated gastric fluid was found. The results were presented in Table 2. Figure 4 show the separation of the combined- tablet mixture solution in simulated gastric fluid with purity confirmation.

### Statistical comparison of the proposed method with reported methods

The results of the assay of AMX, MET, and VPZ in their laboratory prepared combined tablet mixtures obtained by the proposed HPLC–DAD were statistically comparable with reported chromatographic and/or spectrophotometric methods using the student`s t-test and variance ratio F-test (Supplementary Table S6). No reported method was found for AMX, MET and VPZ simultaneous analysis. However, for such comparison, an HPTLC method [25] for analysis of AMX and MET in their ternary mixture with FAM was used to compare our AMX and MET proposed method data while a reported spectrophotometric method [26] for analysis of VPZ was used to compare our VPZ proposed method data. The experimental values did not surpass the theoretical ones, thus indicating the absence of any significant differences between the proposed method and the reported methods.

### Greenness evaluation of the proposed HPLC- DAD method

Nowadays, a menace- free environment is a major aspect of most anthropogenic activities especially, research. Hence, certain methods were adopted to ensure the analytical methods greenness.

The greenness of the proposed HPLC–DAD method was initially evaluated by ESA which revealed a score of 88 (Table 3) that represents sublime green analysis [29].

**Table 3:** Greenness evaluation of the proposed HPLC -DAD method

	Eco-scale assessment		GAPI	NEMI	AGREE
Reported HPLC–DAD method [40]	**Reagents**	**Penalty points**			
	acetonitrile	1 × 4 = 4			
	Sodium dihydrogen	2 × 0 = 0			
	Phosphate				
	**Instrument**				
	Energy	0			
	Occupational hazard				
	Waste	5			
	**Total penalty points**	∑ 10			
	**Eco- scale assessment score**	100—10 = 90			
Proposed HPLC method	**Reagents**	Penalty points			
	Methanol	1 × 6 = 6			
	Sodium dihydrogen	2 × 0 = 0			
	phosphate				
	**Instrument**				
	Energy	1			
	Occupational hazard	0			
	Waste	5			
	**Total penalty points**	∑ 12			
	**Eco- scale assessment score**	100—12 = 88			

A novel technique called GAPI, firstly established in 2018, [31] contributed also to the greenness evaluation of the proposed HPLC–DAD method. GAPI tool rates the greenness of a whole analytical methodology stepwise as it consists of five pentagrams colored with green through yellow to red, as the red color indicates the highest ecological hazards [31]. The obtained GAPI pictogram indicates superb greenness of the proposed HPLC–DAD method as it contains six green zones, seven yellow zones while there are two red zones (Table 3). However, the six green zones represent no preservative (zone 2), no transport (zone 3), no storage (zone 4), no advanced treatment like derivatization (zone 8), the use of hermetic system (zone 13), and waste recycling (zone 15). While, the seven yellow zones represent the inline collection (zone 1), the use of simple procedure like extraction and filtration (zone 5), the use of microextraction (zone 6), the use of green solvents (zone 7), the use of moderately toxic solvent (zone 10), with flammability scale of 2 or 3 (zone 11), and energy consumption of less than 1.5 kwh per sample (zone 12). Finally, the remaining two red zones represent solvents volume greater than100 mL (zone 9) and waste amount more than 10 mL (zone 14).

NEMI tool was also used to assess the greenness of the proposed HPLC–DAD method. NEMI pictogram embraces of four quadrants which indicate the following criteria: non- persistent, bio-accumulative nor toxic (PBT), non- hazardous, nor corrosive reagents, and the method does not generate large amount of waste (more than 50 g per sample analysis). The green color of a NEMI pictogram quadrant indicates the fulfillment of the identified criterion while if the criterion hasn´t been met, the quadrant is left blank [32]. However, methanol contribution in the mobile phase of the proposed method came up with the quadrant representing the hazardous potential of the method to be left blank (Table 3).

Finally, a more overarching presentation of the method greenness was obtained using the recent AGREE tool which was established in 2020. It consists of a circular pictogram of twelve sections colored from intense green to red with a final middle score from 0 (the least greenness) to 1 (the highest greenness) [30]. As for the proposed HPLC–DAD method, eight sections are colored green representing obedience of the following criteria of GAC, on-line analysis (section1), 0.02 mL sample (section 2), in-line device position (section 3), few steps of sample preparation (section 4), automation of the applied method with minimum sample preparation (section 5), no derivatization was carried out (section 6), the analysis of three analytes and nine samples per hour (section 8), using small amounts of toxic reagents (section 11) and the degree of flammability and corrosiveness of the reagents used (section 12). The final score of the proposed method was 0.78 which represents an acceptable environmentally- benign method. while there were two yellow sections representing consuming energy as being a liquid chromatography technique (section 9) and the biological origin of some of the used reagents (section 10). Only one section in the perimeter was colored orange (section 7) as the produced amount of waste was about 30 mL per run.

Ultimately, an integral evaluation of the proposed HPLC–DAD method greenness was established using the greenness assessment approaches including ESA, GAPI, NEMI and AGREE. It is crucial to emphasize that, the results of the greenness appraisal of the proposed method were satisfactory when compared with another previously conducted study of HPLC analysis of another similar mixture composed of AMX, MET, and FAM as a representative example [40].

## Conclusion

The present work described a simple, sensitive and reliable HPLC–DAD method for the analysis of AMX, MET, and VPZ either in their individual tablets, combined-tablet mixtures and the combined tablet mixture in gastric fluid. To our knowledge, this method is the first HPLC–DAD method for the simultaneous determination of the three mentioned drugs.

The developed method was successfully validated as per ICH guidelines and was further applied to the simultaneous analysis of AMX, MET, and VPZ in simulated gastric fluid without any interference.

This work could be readily adopted for the routine quality control of such drugs (AMX, MET, and VPZ) when being co-formulated in single dosage form in future. Besides, it could help to investigate the influence of the three drugs, (AMX, MET and VPZ) on each other in gastric fluid in future studies.

The method is environmentally benign based on the greenness evaluation using the four assessment tools, ESA, NEMI, GAPI and AGREE.

### Supplementary Information

Supplementary Material 1.

## Data Availability

The dataset supporting the conclusions of this article are included within the article and its additional file.

## Abbreviations

AGREE: Analytical Greenness metric
AMX: Amoxicillin
ESA: Analytical eco-scale assesment
GAPI: Green analytical procedure index
HPLC–DAD: High performance liquid chromatography-Diode array detector
H. pylori: Helicobacter pylori
ICH: International Council for Harmonisation of Technical Requirements for Pharmaceuticals for Human Use
MET: Metronidazole
NEMI: National environmental method index
VPZ: Vonoprazan
