# Supplementary material for: An eco-friendly liquid chromatographic analysis of the triple therapy protocol of amoxicillin, metronidazole and vonoprazan for H. Pylori eradication: application to combined dosage forms and simulated gastric fluid
Source: BMC Chem. 2024 May 30;18(1):106. doi: 10.1186/s13065-024-01210-6 (PMC11138008; doi:10.1186/s13065-024-01210-6)
Supplement: Supplementary file 1 — Supplementary Material 1. [file 13065_2024_1210_MOESM1_ESM.docx]

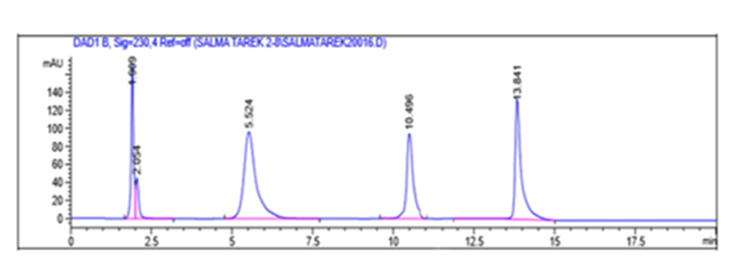


VPZ

MET

AMX


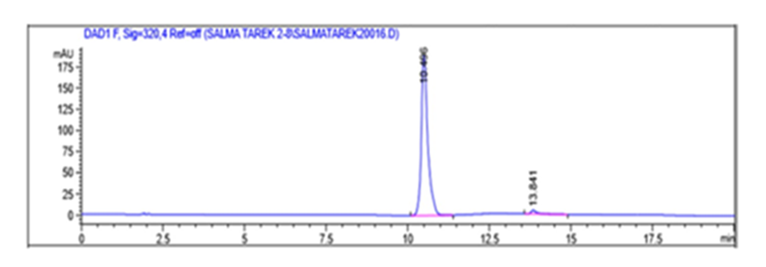


MET

**Sup. Figure 1: Typical HPLC chromatograms of a solution containing 200 µg mL^-1^ AMX, 100 µg mL^-1^ MET and 100 µg mL^-1^ VPZ showing tailed AMX and VPZ peaks using the C18 column and the selected mobile phase (t_R_ = 5.52, 10.50 and 13.84 min, respectively) scanned at 230 nm for AMX and VPZ (a) and 320 nm for MET (b).**

**Sup. Figure 2: Calibration plots of the regression data of peak area and the corresponding concentrations of (a) AMX, (b) MET and (c) VPZ.**

**Sup. Table 1: The gradient elution program used for the separation of AMX, MET and VPZ ternary mixture using the proposed HPLC method.**

| Time  (minute) | % Methanol  (Solvent A)  (v/v) | % Phosphate buffer  (Solvent B)  (v/v) |
| --- | --- | --- |
| 0-6 | 5 | 95 |
| 10 | 50 | 50 |
| 10-15 | 50 | 50 |
| 20 | 5 | 95 |

**Sup. Table 2: Study of different isocratic systems for separation of AMX, MET and VPZ using the proposed HPLC- DAD method.**

| **Trial No.** | **Mobile phase composition (v/ v)^*^** | **Conclusion** |
| --- | --- | --- |
| **1.** | **30% Methanol: 70% Buffer** | - **Fumarate and AMX are not well separated.** - **AMX eluted early.** - **VPZ eluted too late** |
| **2.** | **50% Methanol: 50% Buffer** | - **Fumarate and AMX are not well separated.** - **AMX eluted early.** |
| **3.** | **70% Methanol: 30% buffer** | - **Fumarate, AMX and MET not well separated.** - **All drugs eluted early** |
| **4.** | **40% ACN: 60% buffer** | - **No separation.** - **MET has a forked peak** |
| **5.** | **50% ACN: 50% buffer** | - **No separation of fumarate, AMX and MET** |
| **6.** | **50% Methanol: 30% buffer: 20% ACN** | - **Fumarate, AMX and MET not well separated.** |

**^*^All systems were tried using C-8 column, flow rate 1 ml/min with 30 mM phosphate buffer at pH 5.**

**Sup. Table 3: System suitability parameters for the HPLC-DAD determination of AMX, MET and VPZ ternary mixture.**

| **Parameters** | **AMX** | **MET** | **VPZ** |
| --- | --- | --- | --- |
| **t_R_ ± SD (minutes)** | **3.70 ± 0.15** | **9.35 ± 0.01** | **13.16 ± 0.02** |
| **Capacity factor (K^'^)** | **2.00** | **3.85** | **5.32** |
| **Theoretical plates (N)** | **4270** | **29523** | **40508** |
| **Selectivity (α)** | **1.41** | **1.83** | **1.11** |
| **Resolution (R_S_)** | **3.35** | **11.27** | **3.16** |
| **Asymmetry factor (A_f_)** | **0.92** | **1.01** | **1** |

**Sup. Table 4: Accuracy, intraday and inter-day precision for the determination of AMX, MET and VPZ using the proposed HPLC-DAD method (n=3)**

| **Drug** | **Nominal value (µg mL^-1^)** | | | **Mean % recovery ± SD^a^** | | | **Er(%) ^b^** | | | **Precision % RSD ^c^** | | | | | | |
| --- | --- | --- | --- | --- | --- | --- | --- | --- | --- | --- | --- | --- | --- | --- | --- | --- |
|  |  |  |  |  |  |  |  |  |  | **Intraday** | | | **Inter-day** | | | |
|  | **MIX 1** | **MIX 2** | **MIX 3** | **MIX 1** | **MIX 2** | **MIX 3** | **MIX 1** | **MIX 2** | **MIX 3** | **MIX 1** | **MIX 2** | **MIX 3** | **MIX 1** | **MIX 2** | **MIX 3** |  |
| **AMX** | **600** | **300** | **100** | **99.16 ±0.50** | **98.84 ± 1.13** | **100.73 ±0.93** | **-0.84** | **-1.16** | **0.73** | **0.50** | **1.14** | **0.92** | **0.34** | **1.43** | **0.25** |  |
| **MET** | **200** | **300** | **200** | **98.06 ± 1.50** | **99.77 ± 1.47** | **98.35 ± 1.32** | **-1.94** | **-0.23** | **-1.65** | **1.53** | **1.47** | **1.34** | **1.61** | **0.95** | **1.72** |  |
| **VPZ** | **16** | **100** | **100** | **101.50 ± 0.22** | **99.70 ± 0.76** | **99.70 ± 1.73** | **1.50** | **-0.3** | **-0.3** | **0.22** | **0.76** | **1.74** | **0.60** | **1.16** | **0.77** |  |

**^a^ mean % recovery of three determinations ± SD (n=3).**

**^b^ % relative error**

**^c^ % relative standard deviation**

**Sup. Table 5: Evaluation of the robustness of the proposed HPLC -DAD method for the determination of AMX, MET and VPZ mixture.**

| **Parameters** | **AMX** | | | **MET** | | | **VPZ** | | |
| --- | --- | --- | --- | --- | --- | --- | --- | --- | --- |
|  | **Mean % recovery**  **±SD^a^** | **RSD%^b^** | **t_R_ ± SD^c^** | **Mean % recovery**  **±SD^a^** | **RSD%^b^** | **t_R_ ± SD^c^** | **Mean % recovery**  **±SD^a^** | **RSD%^b^** | **t_R_ ± SD^c^** |
| **Mobile phase ratio**  **± 2% of the first and second ratio^*^** | 100.36 ± 1.23 | 1.23 | 3.70 ± 0.87 | 98.89 ± 0.97 | 0.98 | 9.35 ± 0.52 | 99.90 ± 1.17 | 1.17 | 13.16 ± 0.84 |
| **pH of buffer**  **± 0.2 pH units** | 99.68 ± 0.30 | 0.30 | 3.70 ± 0.01 | 99.13 ± 1.04 | 1.05 | 9.35 ± 0.001 | 100.44 ± 0.63 | 0.63 | 13.16 ± 0.16 |
| **Wavelength**  **± 2 nm** | 100.70 ± 0.55 | 0.55 | 3.70 ± 0.001 | 100.58 ± 1.38 | 1.37 | 9.35 **±** 0.001 | 99.98 ± 0.30 | 0.30 | 13.16 **±**0.001 |

a mean % recovery of the synthetic mixture 100 μg mL^-1^ AMX, 200 μg mL^-1^ MET and 100 μg mL^-1^ VPZ at each experimental parameter (n=3)

b % relative standard deviation

^c^ % relative error

*Start with 7% methanol: 93% buffer then 48% methanol: 52% buffer (gradient) OR start with 3% methanol: 97% buffer then 52% methanol: 48% buffer (gradient)

**Sup. Table 6: Statistical evaluation of the results obtained by the proposed HPLC method and reported methods for the determination of AMX, MET and VPZ in their laboratory prepared combined tablet mixtures.**

|  | **AMX** | | **MET** | | **VPZ** | | |
| --- | --- | --- | --- | --- | --- | --- | --- |
| **Method** | **Proposed HPLC** | **Reported HPTLC [24]** | **Proposed HPLC** | **Reported HPTLC [24]** | | **Proposed HPLC** | **Reported**  **Spectrophotometry [26]** |
| **% Recovery^a^** | 99.17  98.65  100.43  100.32  99.98  99.47 | 99.95  101.33  101.12  98.21  99.21  100.17 | 100.24  101.34  99.58  99.14  99.65  100.08 | 100.51  98.50  98.00  101.21  99.13  98.17 | | 101.13  101.58  100.75  99.30  100.98  99.99 | 99.81  99.95  100.19 |
|  |  |  |  |  | |  |  |
|  |  |  |  |  | |  |  |
|  |  |  |  |  | |  |  |
|  |  |  |  |  | |  |  |
|  |  |  |  |  | |  |  |
| **Mean ± SD^b^** | 99.67 ± | 99.99 ± 1.17 | 100.01 ± 0.76 | 99.25 ± 1.32 | | 100.62 ± 0.83 | 99.98 ± 0.19 |
|  | 0.70 |  |  |  | |  |  |
| **S2^c^** | 0.49 | 1.38 | 0.58 | 1.75 | | 0.69 | 0.04 |
| **t^d^ critical** | 0.59 | | 1.21 | | | 1.27 | |
| **F^d^ critical** | 0.35 | | 0.33 | | | 18.78 | |
